# News

**DOI:** 10.1155/S1463924602000214

**Published:** 2002


Journal of Automated Methods & Management in Chemistry
Vol. 24, No. 6 (November–December 2002) pp. 209–210

### New software

### Fast new crystal data import and moving models enhance INCA Crystal

A fast new method for viewing, importing and editing crystal structures, and the dynamic display of crystal orientation during data acquisition are among the significant benefits now available to users of the INCA microanalysis suite from Oxford Instruments Analytical.

Analytical's PC-based INCA platform is already very highly regarded for its powerful high-accuracy analysis capability and ease of use. New features in the latest issue software (issue 12) offer both new functionality and a number of usability improvements.

Users of INCA Crystal, which offers superb texture and grain boundary analysis by electron backscattered diffraction (EBSD), can now review and import all the crystallographic parameters needed to perform analysis on a given sample, quickly and simply. The new View Phase in INCA Crystal's navigation system directly accesses industry standard '.CIF' crystal structures, which can be used without conversion, and also provides the full range of symmetry information, i.e. space group, etc. for information. The new Edit Phase step allows the import or entry of new structures, either from a database or from printed material, with great ease and flexibility. Both new steps display crystal structures with a highly detailed Crystal Model or 'Mimic'.

This latest version of INCA Crystal also features enhanced colour contour plotting in pole figures and inverse pole figures. Users can, according to preference, position the RD axis at either the top or the side of pole figures. More expert users can also now opt for either equal area or stereographic pole figure plotting methods.

Moving pictures during data collection are also available for those who like to keep one eye on crystal orientation as they acquire data. A Crystal Mimic is now constantly updated in 'real time', so orientation can be viewed as a dynamically changing image—extremely informative in the analysis of deformed materials. The Crystal Mimic is also available in texture analysis—just click on a point and see the orientation.

*For further information, contact Analytical, Halifax Road, High Wycombe HP12 3SE, UK. Tel: +44 (0)1494 442255; Fax: +44 (0)1494 461033; e-mail: analytical@oxinst.co.uk. Internet: http://www.oxford-instruments.com*

### Zymark introduces new system integration software

Zymark Corporation announces the release of CLARA™ 2.2, the third iteration of the CLARA software suite, which continues to build upon its industry leading performance and ease of use. Developed for the scientists' needs, CLARA is an integral part of any successful drug-discovery automation solution.

CLARA incorporates new Plug-and-Play resource adapters to provide a simplified environmental set-up. Any instrument in the automated system is instantly identified by CLARA and ready for drag-and-drop assay creation. CLARA has more than 100 adapters for third-party instruments, giving users an unprecedented degree of system flexibility. In addition, its optimization tools have been enhanced further to improve assay automation and throughput. The well-known application scheduler has been updated increasing computational speed. The new real-time Gantt Chart displays run-time progress and completion of instructions, enabling effective resource utilization.

Assay development and validation is streamlined by one-click simulation of any instrument in the system. Advanced User Notification allows assays to run unattended: cell phones, pagers and e-mail accounts can be contacted in response to resource errors, data milestones or quality assurance statistics.

Available for use with every Zymark Twister® II and integrated system, CLARA 2.2 is available as a new or upgrade package. The software is key to the development of HTS, genomic and proteomic automated solutions.

*For more information, contact Lynda Thomas. Tel: +1 508 497-6549; e-mail: lynda.thomas@zymark.com; Internet: http// www.zymark.com*

### News

### VelQuest partners with Waters

VelQuest and Waters Corporation have announced a marketing partnership and joint effort to provide a seamless interface to the Waters Millennium[32]® Chromatography Data System (CDS) to help scientists manage scientific data better. First introduced in 1992, Millennium[32] was the first of its kind to incorporate fully an Oracle® relational database allowing chemists to locate information effortlessly and spot trends in their chromatographic analyses. The Millennium[32] Interface will allow VelQuest's electronic notebook (ePMC) system to transfer critical sample preparation data electronically using the Millennium[32] Toolkit. ePMC provides a fully validated, paperless approach to data acquisition, documentation, storage and retrieval for use in new product submissions, product releases, stability testing, QA and management analysis.

To date, scientists have struggled with critical applications not effectively sharing information from other applications, and therefore requiring arduous manual input of data. VelQuest offers the interface necessary for the ePMC system to take full advantage of the available features in the Millennium[32] Toolkit while seamlessly allowing the automatic retrieval of critical data such as sample weights, dilution factors, and information on standards and buffers. Also, having the chromatographic file linked to the meta data being used for quantification will greatly facilitate the time for test data and batch record review. The scientist or QA reviewer no longer has to locate the data: they simply click on a box and have the data, methods and all other supporting meta data displayed. Transcription errors due to manual entry of data are also eliminated and productivity gains realized by automatic transfer of data.

The partnership with Waters benefits scientists using Millennium[32] software by providing them with a system for handling non-chromatography instruments in a compatible manner to the Millennium[32] CDS. The combination meets the FDA 21 CFR Part 11 documentation issues while establishing a GxP electronic notebook-based compliance platform they can build on in the future.

The VelQuest ePMC system is aimed directly at managing the productivity of laboratory analysts. It is estimated that approximately 70% of laboratory resources are dedicated to meeting the compliance standards. The ePMC system ensures compliance as well as improving productivity by more than 25%.

*For further information, contact: Sally A. Mitchell, Velquest Corporation, Hopkinton, MA, USA. Tel: + 1 508 497 0128; e-mail: smitchell@velquest.com; Internet: http://www.velquest.com; or Brian J. Murphy, Waters Corporation, Milford, MA, USA. Tel: + 1 508 482 2614; e-mail: brian_j_murphy@waters.com; Internet: http://www.waters.com*

**Fluid cash-flow solution for chemical analysts**

Fortis Commercial Finance has provided a funding solution for an international leader in the field of mercury measurement.

PS Analytical Ltd, the scientific equipment manufacturer and problem solver, and international leader in the field of mercury measurement, has seen substantial growth since turning to Fortis Commercial Finance for funding.

The business was formed in 1983 by the husband-and-wife team of Peter and Margaret Stockwell to manufacture, assemble, and distribute scientific and analytical equipment. The company specializes in the low-level detection of mercury, arsenic, selenium and other harmful chemicals in water, soils, sludge's, effluents and gasses. PS Analytical's customers include major water utilities, oil and gas companies, universities, and chemical companies.

Fortis has worked closely with Peter Stockwell, Managing Director, to match the funding requirements to the company's business goals and objectives. With an annual growth rate of 20% being achieved, the company wanted to investigate more flexible methods of finance in order to keep step with its considerable growth projections in the UK and abroad. The structured invoice discounting facility from Fortis also provides finance and collections in the USA, Finland, Norway, France, Italy and Spain.

Peter Stockwell said,

> Previously having only used overdraft funding, it has been an interesting and highly beneficial experience since using the Fortis Commercial Finance facility. The increased working capital available to set more flexible arrangements with our suppliers, and it has also allowed us to use other funds for our extensive research and development of new products. ... In my view, Fortis stands alone as the only financier that provides such a successful and flexible solution that funds the debt for our operations throughout the world. Working together as equal partners, the Fortis facility has helped our business overcome the financial and cultural problems associated with trading overseas.

PS Analytical now has a significant market share in the UK and continues to be committed to research and development, building up strong links with universities and other institutions through the loan of equipment for research purposes. Over the past 5–6 years, the company has worked with groups around the world to establish reliable measurements of mercury levels in fossil fuels. In particular, it has worked on new applications to measure chimneystack emissions and analyse natural gas for mercury content.

*For further information, contact: PS Analytical Ltd, Arthur House, Unit 3, Crayfields Industrial Estate, Main Road, St Pauls Cray, Orpington BR5 3HP, UK. Tel: + 44 (0)207 535 1355; Fax: + 44 (0)207 535 1350; e-mail: pbs@psanalytical.demon.co.uk*

